# The Swallowing Characteristics of Thickeners, Jellies and Yoghurt Observed Using an In Vitro Model

**DOI:** 10.1007/s00455-019-10074-1

**Published:** 2019-11-09

**Authors:** Simmi Patel, William J. McAuley, Michael T. Cook, Yi Sun, Shaheen Hamdy, Fang Liu

**Affiliations:** 1 Department of Clinical and Pharmaceutical Sciences, School of Life and Medical Sciences, University of Hertfordshire, Hatfield, AL10 9AB UK; 2 Department of Engineering and Computer Science, University of Hertfordshire, Hatfield, AL10 9AB UK; 3 GI Sciences, Division of Diabetes, Endocrinology and Gastroenterology, Faculty of Biology, Medicine and Health, University of Manchester, Salford Royal Hospital, Salford, M6 8HD UK

**Keywords:** Dysphagia, In vitro, Oral transit, Rheology, Swallowing, Texture, Deglutition, Deglutition disorder

## Abstract

Drinks and foods may be thickened to improve swallowing safety for dysphagia patients, but the resultant consistencies are not always palatable. Characterising alternative appetising foods is an important task. The study aims to characterise the in vitro swallowing behaviour of specifically formulated thickened dysphagia fluids containing xanthan gum and/or starch with standard jellies and yoghurt using a validated mechanical model, the “Cambridge Throat”. Observing from the side, the model throat can follow an experimental oral transit time (in vitro-OTT) and a bolus length (BL) at the juncture of the pharynx and larynx, to assess the velocity and cohesion of bolus flow. Our results showed that higher thickener concentration produced longer in vitro-OTT and shorter BL. At high concentration (spoon-thick), fluids thickened with starch-based thickener showed significantly longer in vitro-OTT than when xanthan gum-based thickener was used (84.5 s ± 34.5 s and 5.5 s ± 1.6 s, respectively, *p* < 0.05). In contrast, at low concentration (nectar-like), fluids containing xanthan gum-based thickener demonstrated shorter BL than those of starch-based thickener (6.4 mm ± 0.5 mm and 8.2 mm ± 0.8 mm, respectively, *p* < 0.05). The jellies and yoghurt had comparable in vitro-OTT and BL to thickeners at high concentrations (honey-like and spoon-thick), indicating similar swallowing characteristics. The in vitro results showed correlation with published in vivo data though the limitations of applying the in vitro swallowing test for dysphagia studies were noted. These findings contribute useful information for designing new thickening agents and selecting alternative and palatable safe-to-swallow foods.

**Electronic supplementary material:**

The online version of this article (10.1007/s00455-019-10074-1) contains supplementary material, which is available to authorised users.

## Introduction

Dysphagia, characterised by a difficulty in swallowing, is a complication associated with several conditions, including stroke, dementia and neurological disorders [1]. An ageing population means there is an increased interest in this condition [2, 3]. Without treatment, dysphagia can lead to food avoidance, particularly in the elderly, which may result in serious consequences such as nutritional and respiratory complications, and even death [4, 5].

Food consistency and texture impact swallowing safety. Viscosity, cohesiveness, hardness and adhesiveness affect the physical nature of the bolus and how it moves within the aerodigestive tract [6]. Adding thickeners to drinks and food is a popular management strategy for dysphagia. Thin liquids are difficult for these patients to consume because of their low resistance to flow, which may cause them to spill out of the mouth or over into the airway leading to aspiration. Thickeners increase the viscosity of thin drinks and food stuffs making them easier and safer to swallow [6]. Increasing bolus viscosity helps the bolus to remain in the oral phase for longer [7], giving the patient more time for reflex responses and muscular adjustments essential for safe swallowing [8].

Whilst the thickening of drink and food is common practice, there is an ongoing debate within the healthcare profession about the optimum fluid consistency [6]. Very thick liquids and solid foods may leave a residue in the throat or need greater propulsion from the tongue to drive material through the oropharynx [6]. In addition, many of these thickened liquids are poorly accepted by patients [9, 10]. Other foodstuffs, such as jellies and yoghurts, maybe more palatable, but their swallowing characteristics have not been fully established.

Dysphagia can be monitored using in vivo techniques, like fiberoptic endoscopic evaluation of swallowing (FEES) and videofluoroscopic swallowing study (VFFS), but these methods are linked with significant drawbacks, including the fact they are invasive, as well as costly and cumbersome [11, 12]. Therefore, quantifiable, non-invasive or in vitro methods for assessing the suitability of different food and liquid textures in dysphagia management are of great interest, resulting in some key developments in this field. Mackley et al. developed a mechanical device, an in vitro swallowing simulator, for tracking the swallowing characteristics of different fluids [7], which was further improved by Hayoun et al. [3]. Mowlavi et al. extended this work by further characterising the mechanics of the throat model with validation achieved through in vivo observations [13].

The in vitro swallowing simulator provides a tool that imitates some aspects of in vivo swallowing to enable the comparison of the flow behaviour of different types of fluids. Using this model, Mowlavi et al. described the two distinct phases of the bolus movement during the oral phase of swallowing: the initial acceleration proportional to the applied force and system inertia followed by the viscous regime whereby the bolus velocity is governed by its viscosity [13]. The effect of the applied force on bolus flow is relevant to dysphagia patients who might experience low tongue pressure. The mechanical model also looked at mechanisms for applying shear-thinning fluids in the treatment of dysphagia [13]. Previous studies applied the in vitro swallowing simulator to characterise the bolus velocity or transit time of the fluids in the model [3, 7, 13]. However, it is also important to consider the cohesiveness of bolus flow which prevents spillage into the larynx [4].

In this study, we applied the original swallowing simulator [7] to explore the suitability of using the in vitro model to characterise the flow behaviour of fluids and soft foods for dysphagia management. The study aimed to achieve the following objectives using the in vitro model: (1) quantify the bolus flow in the in vitro model using the in vitro-oral transit time (in vitro-OTT) and bolus length (BL) at the juncture of the pharynx and larynx to assess the velocity and cohesiveness of the bolus flow, respectively; (2) characterise the swallowing performance of commonly used thickeners, including those containing xanthan gum and starch, at different consistencies; (3) evaluate the suitability and limitations of applying the in vitro model in dysphagia in light of the existing in vivo data of thickened fluids; and (4) compare the in vitro swallowing characteristics of alternative foods, including jellies and yoghurt, with thickening agents.

## Materials and Methods

### Materials

Three thickeners, five jellies and a smooth yoghurt, all containing commonly used gelling agents, were evaluated (Table 1).

**Table 1:** Thickening agents, jellies and yoghurt used in the study alongside the gelling agents and product manufacturer (supplier)

Product	Gelling agents	Manufacturer (supplier)
Thickeners		
Thick & Easy®^a^	Modified starch	Fresenius Kabi, Ireland (ASDA supermarket, UK)
Resource® ThickenUp^TM^ Clear^a^	Xanthan gum	Nestle Health Science, Switzerland (ASDA supermarket, UK)
Nutilis powder	Modified starch, xanthan gum, tara gum, guar gum	Nutilis, The Netherlands (ASDA supermarket, UK)
Jellies		
Hartley’s™ strawberry ready-to-eat jelly^a^	Locust bean gum, xanthan gum, gellan gum	Hain Daniels Group, UK (ASDA supermarket, UK)
Vimto™ ready-to-eat jelly^a^	Carrageenan, locust bean gum	Caterers choice Ltd., UK (ASDA supermarket, UK)
Peppa Pig™ ready-to-eat jelly^a^	Gelatine	Heaven made foods Holt Ltd, UK (ASDA supermarket, UK)
Ryukakusan™ “magic” jelly for adults^a^	Agar	Ryukakusan, Japan (Amazon.com, USA)
Ryukakusan™ “magic” jelly for children^a^	Agar	Ryukakusan, Japan (Amazon.com, USA)
Yoghurt		
Ski® strawberry yoghurt^a,b^	Milk, rice starch, sugar, lemon juice, carrot concentrate, guar gum, milk calcium concentrate	Nestle, Switzerland

*UK* United Kingdom, *USA* United States of America

^a^Referred to as Thick & Easy, Resource Clear, Hartley’s jelly, Vimto jelly, Peppa Pig jelly, Ryukakusan jelly and yoghurt, throughout this paper

^b^Rice starch and guar gum are gelling agents in yoghurt; however, other ingredients contribute to the overall thickness of the yoghurt. Therefore, all ingredients in yoghurt are listed

### Description of the In Vitro Swallowing Simulator

**Fig. 1:**
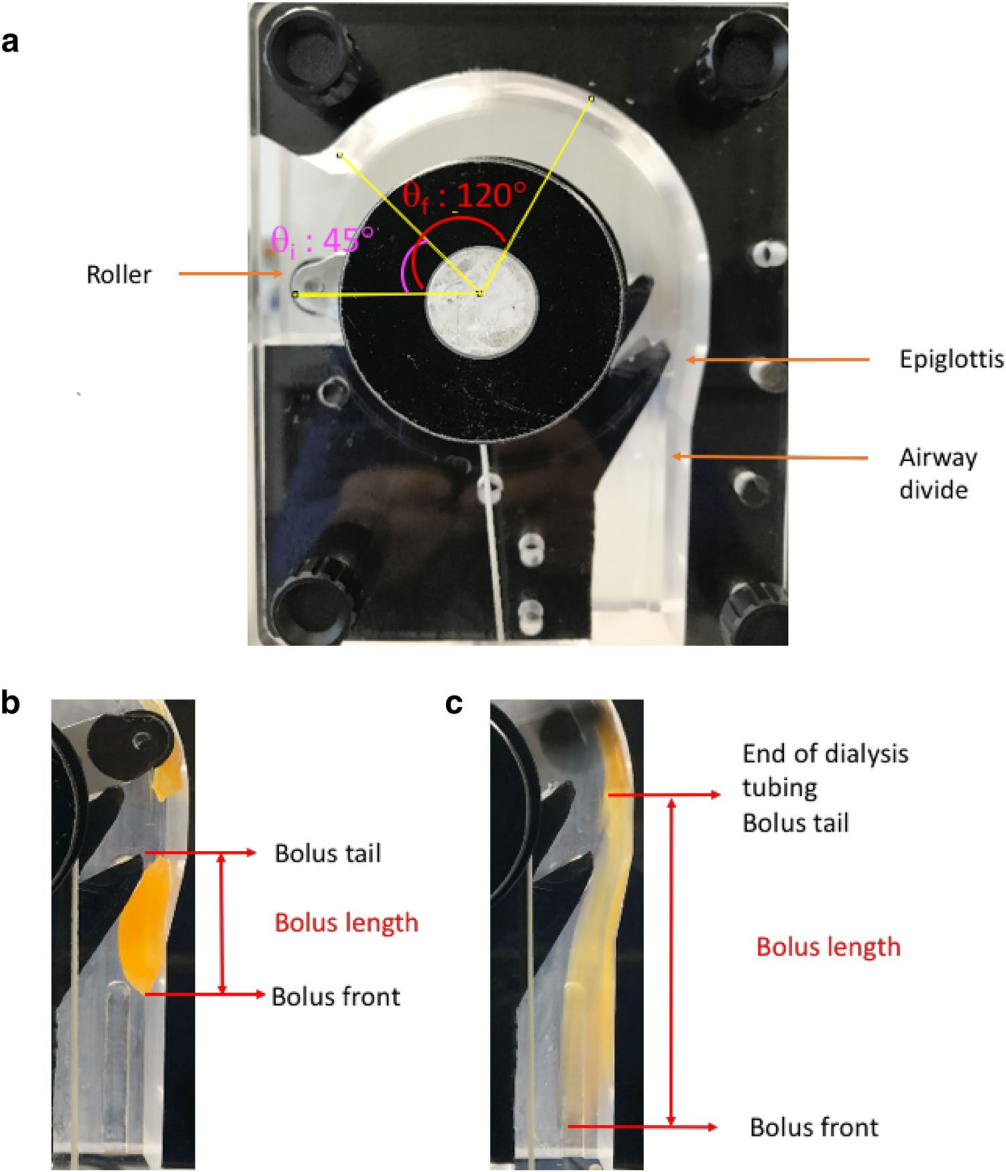
Image of the Cambridge Throat simulator (**a**), and example images showing how bolus length was measured **b** when the bolus tail was clearly seen and **c** when the bolus tail was not clearly seen

The In Vitro Swallowing Simulator—“Cambridge Throat”—is a static mechanical model designed to simulate the physiological anatomy and dimensions of the human throat (Fig. 1) [7]. The test sample (5 mL) was held within a 25 mm wide dialysis tube attached to the curved top of the model, which represented the mouth (Fig. 1). The tongue action was simulated by a roller with an attached weight (190 g) held by a pin. When the pin was released, the weight pulled the roller, which in turn applied pressure on the bolus, moving it through the tubing [7]. The roller movement ended just before the area of the model representing the epiglottis. After this point, the sample flowed under gravity, to a diversion in the model cavity which represented the juncture of the pharynx and larynx, before exiting the tubing (Fig. 1a).

An iPhone camera (6S, Apple, USA) captured images of the test sample flowing through the throat model at 30 frames per second. The in vitro*-*OTT and BL of the test sample at the pharynx and larynx juncture were calculated from the images. In vitro-OTT was calculated as the time taken by the roller to reach an angle of 120° as reported by Mowlavi [13]. ImageJ (Fiji) image processing software was used to calculate BL by capturing the first image in which the bolus front reached the pharynx and larynx juncture. BL was measured as the length of the bolus from front to tail (Fig. 1b). In cases where the bolus tail was not clearly seen, BL was measured from the end of the dialysis tubing to front (Fig. 1c) to provide a standardised measurement.

The in vitro model was designed to mimic the approximate profile of the life-sized human throat [7]. The movement of the roller pulled by the released weight simulated the tongue pressure of approximately 10 kPa [7]. Although this pressure is broadly consistent with reported in vivo measurements [13, 14], it does not account for the variation of oral pressure in relation to bolus consistency. The in vitro-OTT defined by Mowlavi provided satisfactory agreement with in vivo-OTT of Newtonian and shear-thinning liquids observed in human subjects [13]. This standardised calculation may provide a useful comparison between different types of fluids; however, their representation of the physiological oral transit needs to be further evaluated. The epiglottis and the juncture of the pharynx and larynx were included in the model; however, because they are static, they cannot represent the physiological condition whereby the epiglottis closes and protects the entrance of the larynx from bolus entry. In addition, the passage of the bolus through the oesophagus in the in vitro model was driven by gravity, so differed from the in vivo peristaltic oesophageal transit.

### Preparation of Test Samples

Three commercial dysphagia thickeners were used: Thick & Easy (starch-based), Resource Clear (xanthan gum-based) and Nutillis Powder (containing a mixture of starch and gum). The thickeners were each prepared to three consistency levels using the lowest amount of powder recommended by the manufacturers for each consistency (Table 2). The consistency levels were selected according to the National Dysphagia Diet (NDD) guideline recommendations for dysphagia [15] which have been superseded by the International Dysphagia Diet Standardisation Initiative (IDDSI) framework [16]. Whilst the NDD guideline recommends the consistency levels based on a the viscosity range measured at 50 s^−1^, the IDDSI classifies fluid consistency at five levels, 0 to 4 (thin, slightly thick, mildly thick, moderately thick and extremely thick) using the syringe flow test. The IDDSI thickening levels were not tested in this study. Deionized water (100 mL) was used to standardise preparation and minimise the effect of water quality on thickness.

**Table 2:** The amount of thickening content in deionized water at each thickening level

Product	Level of thickening	Thickener content in deionized water % (w/v)
Thick & Easy	Nectar-like	4.5
	Honey-like	6.75
	Spoon-thick	9
Resource clear	Nectar-like	1.2
	Honey-like	2.4
	Spoon-thick	3.6
Nutilis powder	Nectar-like	2
	Honey-like	4
	Spoon-thick	6

w/v: weight/volume

The Ryukakusan jellies and yoghurt were tested in the in vitro swallowing simulator directly from the packages. A spoon was used to remove the yoghurt sample from the package and onto the test plate or into the test tube; yoghurt samples were not stirred. The Hartley’s, Vimto and Peppa Pig jellies were firm, retained a free-standing structure when left on a plate and do not flow. Therefore, they were either manually chopped to particles of 4 mm diameter or chewed by volunteers before the in vitro swallowing test. Healthy volunteers (*N* = 12, aged 29–44 years, five females and seven males) were recruited and each participant chewed one pack each of the three jellies. The participant put a spoonful of the jelly in their mouth, masticated and expelled the bolus when they deemed it ready for swallowing. The temperature and particle sizes of 20 randomly selected particles of the expelled boluses were measured immediately after expulsion using a digital thermometer and a digital calliper (DML 150 mm, Digital Micrometers Ltd). For each of these three jellies, boluses containing similar particle sizes from different participants were mixed together and used for the in vitro swallowing test.

### Rheological and Textural Characterisation

Rheological and textural characterisation of the thickened fluids, jellies and yoghurt were conducted using samples prepared as described above, with the free-standing jellies used after being chopped (dry) or as chewed boluses. A TA 1500 EX controlled-stress rheometer (TA instruments Ltd, United Kingdom) measured steady-shear viscosity and oscillatory viscoelastic data. All measurements were performed at 25 °C using a parallel plate geometry (diameter 40 mm, gap 650 µm). For each sample, an oscillatory stress sweep (torque 0.01–10,000 µNm at a frequency of 10 rad s^−1^) and steady-state rate sweep (0.01–100 s^−1^) were conducted in triplicate. Apparent viscosity was measured at a shear rate of 50 s^−1^ during a steady-state rate sweep and yield stress was measured as the linearity limit of G’ from the stress sweeps. Apparent viscosity was only calculated for thickened fluids.

Textural characteristics were evaluated using a texture analyser (TA.XT. *Plus,* Stable Microsystems, United Kingdom) by applying the back extrusion tests with a 5 kg load cell. An extrusion disc (35 mm) was positioned centrally over the container holding 100 mL of sample so the disc penetrated the sample to a depth of 20 mm at a 0.5 mm s^−1^ test speed. The maximum force (g) used to reach this depth was the measurement of firmness. The maximum negative force, when the probe was drawn up at a speed of 0.5 mm s^−1^, was the indication of cohesiveness. Surface adhesion was determined by drawing the disc at a speed of 0.5 mm/s towards the sample; the disc was then held on the surface of the sample for 30 s and pulled away at 2 mm s^−1^. The force (g) for withdrawal of the disc from the sample indicated surface adhesion (adhesiveness). All measurements were carried out at room temperature and in triplicate.

### Data Analysis

The in vitro-OTT and BL were presented as mean ± 95% confidence interval (CI), calculated using Eq. 1 [17]:

1 $$ {\text{CI}} = {\text{Mean }} \pm 1.96 \left(\sigma/\sqrt{n}\right)$$

Prism Graphpad (Version 7.0) was used to assess the normality of the in vitro-OTT and BL data using the Shapiro–Wilk test—normal distribution was rejected when *p* < 0.05. The Mann–Whitney *U* test was applied to determine significant differences for OTT and BL between different samples; significant differences were noted at *p* ≤ 0.05.

Prism Graphpad was also used to obtain the Pearson correlation coefficient for the in vitro-OTT and BL against rheological (apparent viscosity and yield stress) and textural parameters (cohesiveness, firmness and adhesiveness) of the thickened fluids. The correlation coefficient was graded according to Table 3 [18]. Nine products were included in the calculation (three thickeners at three thickening levels). For each product, the in vitro swallowing performances were measured in five repetitions and the rheological/textural parameters were measured in triplicate (due to low data variation observed). During data analysis, to match the rheological/textural data to the in vitro swallowing data, each measure of the triplicate rheological/textural parameters was randomly allocated an in vitro-OTT or BL from the five in vitro swallowing measurements, which generated 27 samples (triplicate data set for nine products) for the correlation coefficient calculation. To evaluate the reproducibility of the calculation, this calculation was repeated for 100 runs (with random allocation of the five in vitro-OTT/BL measures to the three rheological/textural measurements) and the mean ± standard deviation of the 100 correlation coefficients were reported.

**Table 3:** Correlation coefficient classification [18]

Classification	Correlation coefficient (*R*)
Very high positive or negative correlation	± 0.9 to ± 1
High positive or negative correlation	± 0.7 to ± 0.9
Moderate positive or negative correlation	± 0.5 to ± 0.7
Low positive or negative correlation	± 0.3 to ± 0.5
Negligible correlation	0.0 to ± 0.3

## Results

### In Vitro Swallowing Performance of Thickeners

**Fig. 2:**
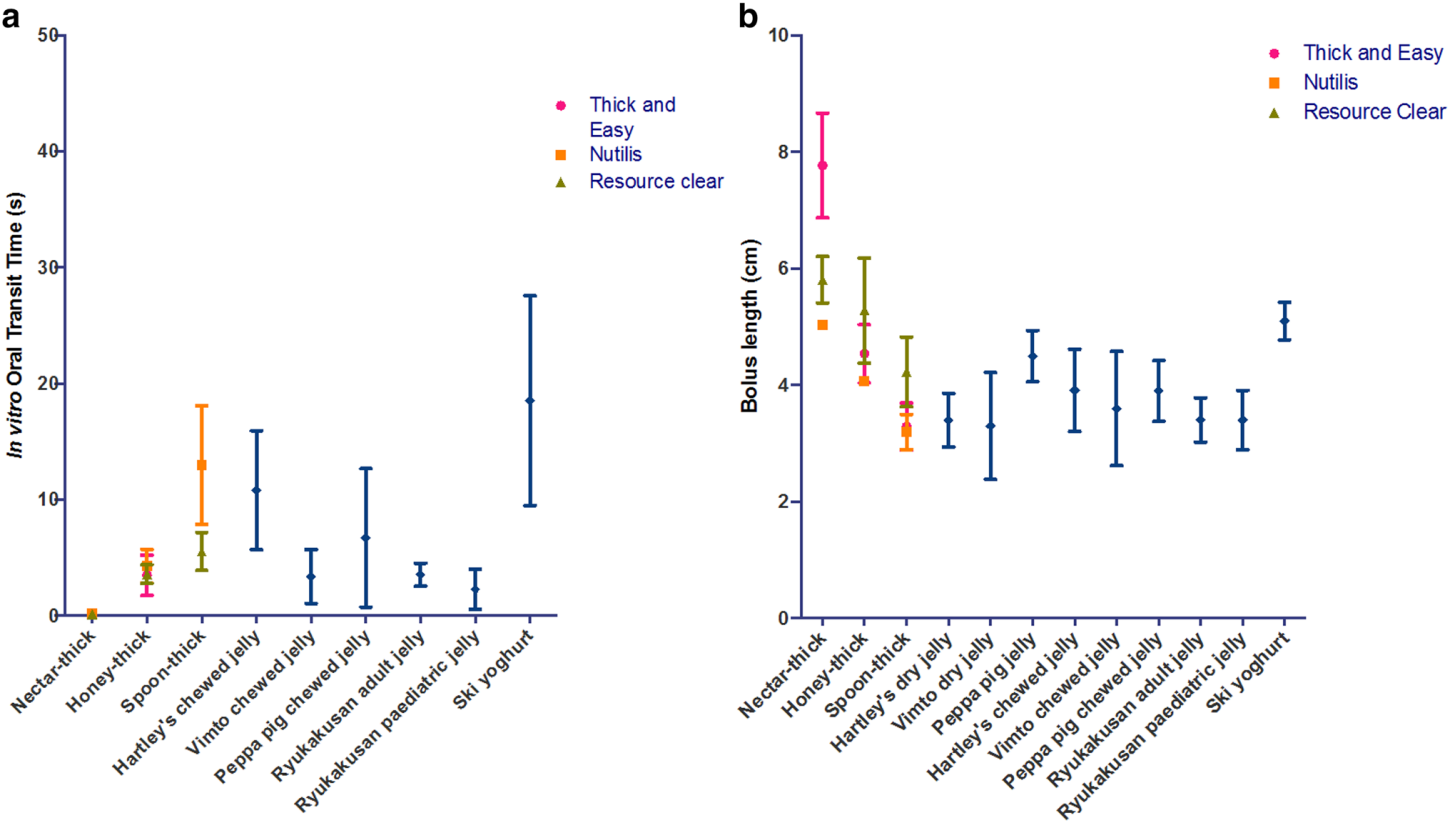
Graphs showing **a** oral transit time and **b** bolus length of the commercial thickeners at nectar-like, honey-like, and spoon-thick thickening levels and jellies and yoghurt

The in vitro-OTT lengthened and BL shortened as concentrations of the thickening agents increased (Fig. 2). Thick & Easy at spoon-thickness produced an in vitro-OTT over 100 s (data not shown). Significant differences in in vitro-OTT and BL were observed between the levels of thickening of each thickener (*p* < 0.05), except for Resource Clear for which there were no significant differences between honey-like and spoon-thick concentrations. The three thickeners showed similar in vitro-OTT at low concentrations (nectar-like and honey-like), but showed significant differences at spoon-thick concentration (*p* < 0.05), with a ranking order of Thick & Easy > Nutilis Powder > Resource Clear. For BL, there was no significant difference between the thickeners at honey-like and spoon-thick concentrations, but at nectar-like concentration, significant differences (*p* < 0.05) were detected with a ranking order of Thick & Easy > Resource Clear > Nutilis Powder.

### In Vitro Swallowing Performance of the Jellies and Yoghurt

The in vitro-OTT of the manually chopped (dry) jellies were over 100 s (data not shown) and significantly longer (*p* = 0.0159 [Hartley’s], 0.0079 [Vimto and Peppa Pig]) than the chewed boluses of the same jelly (Fig. 2). The dry jellies showed slow transit comparable to spoon-thick Thick & Easy. The in vitro-OTT of the chewed jellies and the free-flowing Ryukakusan jellies were comparable to honey-like thickened fluids, whilst Ski yoghurt showed similar in vitro-OTT to spoon-thick Nutilis Powder. BL was not significantly different between dry and chewed jellies for the free-standing jellies. All jelly boluses had a BL between that of honey-like and spoon-thick thickened fluids; however, Ski yoghurt had a longer BL than the jelly samples and was between that of nectar-like and honey-like fluids. The mean temperatures of the chewed free-standing jelly boluses were 24.6 °C (Hartley’s), 25.2 °C (Vimto) and 21.0 °C (Peppa Pig) similar to the room temperature (21.1 °C) at the time of testing. The average particle sizes for the expelled jelly boluses were between 4.3 and 7.2 mm.

### Viscosity and Textural Characteristics of Thickeners, Jellies and Yoghurt

**Fig. 3:**
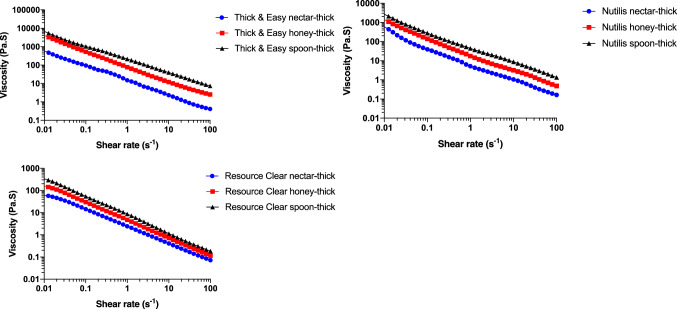
Apparent viscosity as a function of shear rate for thickeners

**Fig. 4:**
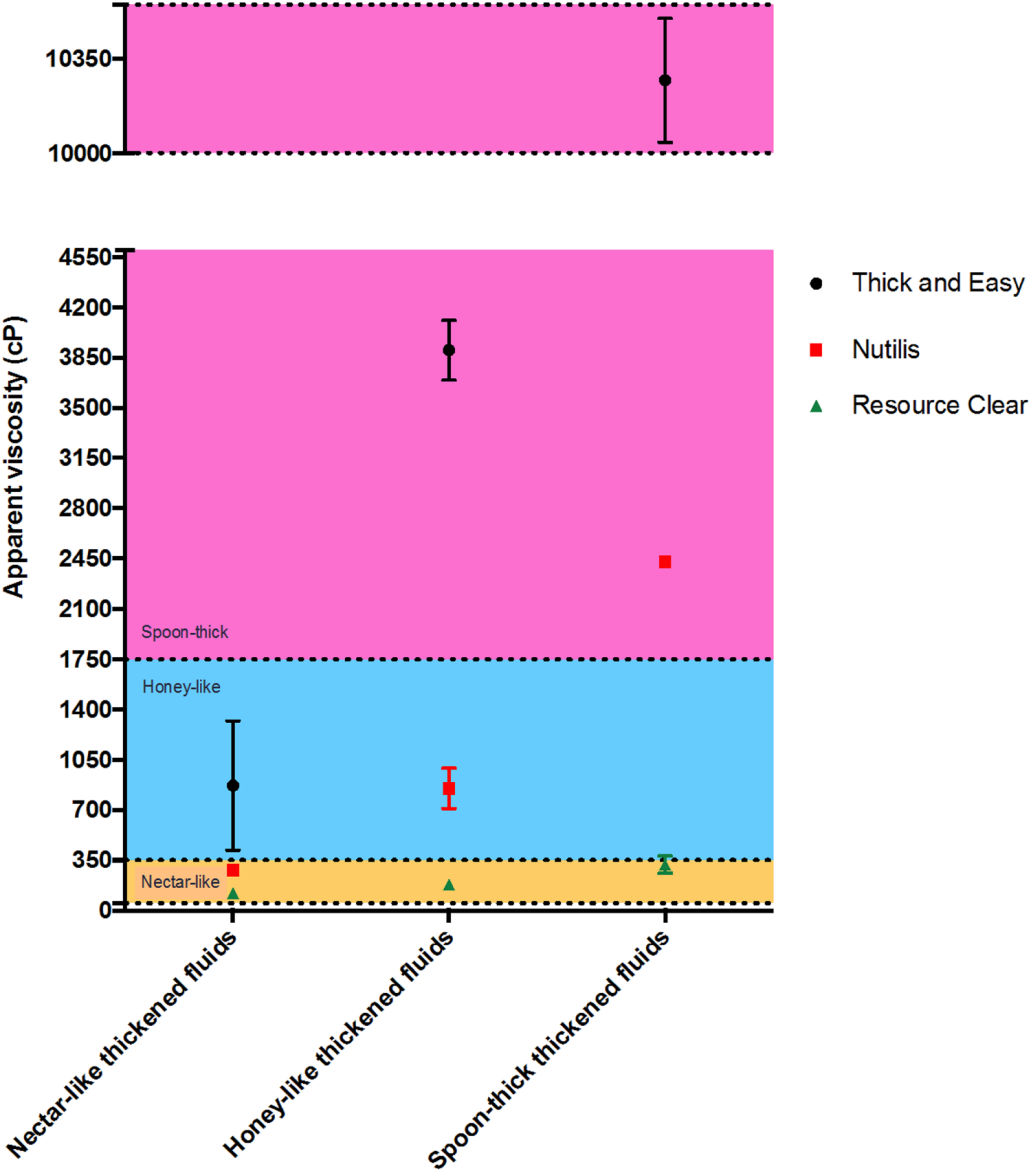
Apparent viscosity (at 50 s^−1^) plotted for thickeners. The expected apparent viscosity ranges for each stage of thickening, in accordance with the National Dysphagia Diet (NDD) guideline recommendations for dysphagia [15], are presented using the coloured bands

Within the three thickeners, Resource Clear had a consistently low viscosity over the shear rate range tested and Thick & Easy had the highest viscosity (Fig. 3). It is to be noted that the measured apparent viscosities at 50 s^−1^ of the thickeners, prepared according to the manufacturers’ recommendations, were different to the expected stages of thickening according to the NDD recommendations for dysphagia (Fig. 4). The apparent viscosities at 50 s^−1^ for Resource Clear (xanthan gum-based) were lower than the expected levels at honey-like and spoon-thick concentrations. Thick & Easy (starch-based) had a higher viscosity than the expected range at nectar-like and honey-like concentrations, and was also in the high end of the expected range for spoon-thick concentration. Nutilis Powder, a mixture of starch and gum-based thickener, had a viscosity within the expected range at all three thickening levels.

Very high or high positive correlations were found between in vitro-OTT and some of the rheological and textural properties of the thickened fluids, including apparent viscosity, yield stress, cohesiveness and firmness (Table 4). Adhesiveness showed negligible correlation with in vitro-OTT. Moderate negative correlations were found between BL and all five rheological and textural parameters; however, high correlation coefficients were noted for cohesiveness and firmness in comparison to apparent viscosity, yield stress and adhesiveness (Table 4).

**Table 4:** Pearson’s correlation coefficients between in vitro-OTT, BL and rheological/textural parameters

Parameters	Correlation with in vitro-OTT		Correlation with BL	
	*R*	Correlation	*R*	Correlation
Apparent viscosity	0.90 ± 0.03	High positive	− 0.52 ± 0.03	Moderate negative
Yield stress	0.97 ± 0.02	Very high positive	− 0.54 ± 0.03	Moderate negative
Cohesiveness	0.88 ± 0.02	High positive	− 0.67 ± 0.03	Moderate negative
Adhesiveness	0.04 ± 0.03	Negligible	− 0.58 ± 0.03	Moderate negative
Firmness	0.80 ± 0.02	High positive	− 0.67 ± 0.04	Moderate negative

## Discussion

In this study, we applied the in vitro swallowing simulator to a range of thickened fluids used for dysphagia treatment, and soft foods. We used a combination of in vitro-OTT and BL to quantitatively characterise the swallowing behaviour of these materials. In vitro-OTT is associated with bolus flow velocity in the throat model. In agreement with published data [7, 13], we found that increasing thickener content increased the in vitro-OTT, which corresponded to an increase in apparent viscosity in the test shear rate range. Comparing the three thickeners, the xanthan gum-based thickener increased the in vitro-OTT to the least extent even at a high concentration (spoon-thick). This agreed with published findings for which that xanthan gum solutions travelled at high velocity in the in vitro model similar to that of low-medium viscosity Newtonian fluids [7, 13]. In contrast, the starch-based thickener had a long in vitro-OTT, especially when spoon-thick, which matched previous observations [7]. As Mowlavi described, the initial movement of the bolus in the in vitro model is predominantly controlled by the system inertia and it is only at the subsequent viscous flow regime that bolus transit is influenced by viscosity [13]. Consequently, the in vitro-OTT of shear-thinning liquids is determined by their high shear rate viscosity (e.g. at 50 s ^−1^) [13], which was much higher for the starch-based thickener compared to the xanthan gum-based thickener at high concentration (spoon-thick). We found that the apparent viscosities of the thickeners, prepared following the manufacturers’ recommended methods, did not always correspond to the expected level of thickening according to the NDD recommendations for dysphagia. Variations in viscosity of commercial thickeners and discrepancies in relation to the NDD standard have been previously reported [19, 20]. Several factors can affect the viscosity of thickened liquids, including the type of thickening agents, media used, e.g. water, juice and milk, solid content, and thickening time [21, 22]. Distilled water was used as the medium in this study, which might be a contributing factor to this result. Therefore, the differences in the in vitro-OTT between xanthan gum- and starch-based thickeners need to be interpreted in light of their different apparent viscosities at 50 s^−1^.

The variations of the measured apparent viscosity at 50 s^−1^ for the thickeners provided the data range to evaluate the correlation between the in vitro-OTT tested in the swallowing simulator and apparent viscosity (at 50 s ^−1^) and a high correlation was established between the two. Cohesiveness, yield stress and firmness also showed good correlation to the in vitro-OTT, which can be attributed to the fact that these properties are associated with the binding force between particles in a bolus, which in turn, affects bolus flow [6, 23, 24].

When using the modified swallowing simulator, Mowlavi noted frequent spillage in the initial oral phase of the in vitro transit of Newtonian liquids boluses, but this phenomenon did not occur in shear-thinning fluids [13]. The authors suggested that this was because shear-thinning liquids had more controlled flow before the swallowing is initiated. Using the in vitro simulator, we found that at low thickener concentration (nectar-like), the xanthan gum-based agent produced a significantly shorter BL compared to the starch-based product, despite their low apparent viscosity. This can be explained by the difference in extensional flow of starch and xanthan gum solutions. During extensional deformation, starch solutions stretch in a non-homogenous manner leading to premature filament break up, whilst xanthan gum-based solutions deform uniformly with extended filament thinning [7]. At a similar shear viscosity, xanthan gum showed higher extensional viscosity compared to the starch-based thickener [25, 26]. The microstructure of the materials further explains this difference: xanthan gum solutions contain dissolved polymer and have a mesh-like structure, whilst starch-based thickeners comprise swollen starch granules [26].

Our in vitro findings have some similarities with those of some existing in vivo studies. For example, low oral velocity of a bolus reduces aspiration and penetration in patients with dysphagia as reported in several in vivo studies [27–31]. This reduction in bolus velocity is usually achieved by increasing the bolus viscosity [29, 32]. For example, a viscous paste bolus was found to increase oral/pharyngeal transit times and overall swallowing duration compared to a low-viscosity liquid bolus in healthy subjects [33, 34]. The benefit of low bolus transit velocity in improving swallowing safety was thought to be due to the provision of extra time for the pharyngeal swallowing response, particularly given that one of the most common causes of aspiration is delayed triggering of the pharyngeal swallow in patients, especially older patients with dysphagia [35]. However, some materials tested in our study showed too long an in vitro-OTT; for example, Thick & Easy had an in vitro-OTT over 100 s at high consistency (spoon-thick), which clearly did not represent the physiological OTT. In vivo, there would be multiple swallows and the long oral clearance may increase post-deglutitive oropharyngeal residue which then causes aspiration after swallowing, a phenomenon associated with starch-based thickeners and especially in patients with reduced muscle strength and deficient bolus propulsion such as those who are elderly [30, 32, 36].

We used BL to indicate the cohesiveness of the bolus flow and observed moderate correlation with cohesiveness and firmness of the thickeners. The cohesive manner of the bolus transit, i.e. as one homogenous bolus without fragmentation, is needed to prevent spillage into the larynx [4], a phenomenon that is less understood compared to the effect of bolus velocity on swallowing safety. The acoustic sound of xanthan gum solutions during pharyngeal swallowing was found to shift to a higher frequency range with increasing concentration [37, 38], which may indicate the presence of “coherent flow” in which the thickener solution flows as one coherent bolus through the pharyngeal phase. In our study, BL decreased as the thickener content increased, which may offer another explanation for the positive effect observed clinically when using thickeners at high consistency to reduce the risk of aspiration in dysphagia patients [28, 39]. The shorter BL of the xanthan gum-based thickener at low concentration compared to the starch-based thickener correlates with results of previous studies using in vivo videofluoroscopic assessment in patients with dysphagia, whereby xanthan gum and starch-based thickeners showed similar efficiency in improving swallowing safety at high viscosity (spoon-thick); however, at low (nectar-thick) viscosity, thickeners containing xanthan gum were more effective in reducing aspiration than those with starch [28, 30]. It is to be noted that the cohesive flow observed in the static in vitro simulator with an open epiglottis cannot be directly extrapolated to in vivo performance due to the discrepancy of the model design to physiological conditions. However, the findings may offer a simple screening tool for different materials before the swallowing safety can be confirmed using in vivo tests.

We found that the in vitro swallowing behaviour of jellies and yoghurt were comparable to honey-like and spoon-thick thickened fluids. The microstructure of yoghurt comprises a protein network with embedded aggregates of casein micelles and fat globules [40]. Jellies are sometimes not distinctive from gels, forming three-dimensional networks of physically cross-linked polymers containing solvent. These structural characteristics could contribute to their in vitro swallowing characteristics. These findings may suggest some advantages of using these types of food in dysphagia patients compared to thin liquids. Yoghurt showed a low risk in aspiration in dysphagia patients because of its cohesive flow through the pharynx previously demonstrated using in vivo acoustic analysis [38]. However, it needs to be noted that prolonged OTT, as indicated by the long in vitro-OTT of “dry” jellies, might increase oropharyngeal residue, though we found that saliva lubrication significantly reduced the in vitro-OTT of the free-standing jellies when chewed, which could mitigate this risk.

To summarise, the quantitative evaluation of the in vitro swallowing characteristics, as reported in this study, showed some similarities with the reported in vivo data. However, the in vitro swallowing model does not mimic the full complexity of in vivo swallowing with respect to chewing, varying oral pressure and the physiological conditions of epiglottis movement and closure. Therefore, our findings cannot be directly translated into in vivo swallowing safety. However, in our chewing test, the jellies were mixed with saliva and digestive enzymes, which provides a closer representation to the in vivo process and affected the in vitro swallowing process, i.e. reduced in vitro-OTT. Due to the differences of the interior surface of the test tube to the physiological oropharyngeal surfaces, it would be useful to evaluate the suitability of testing “dry” (without saliva) samples in the in vitro test. The temperature of the jellies, after chewing, varied by approximately 3 °C, which may impact on viscosity and swallowing. Deionised water was used in this study and the previously reported sample preparations for the in vitro testing [7, 13], but this does not represent normal clinical experience. Further studies are now needed to improve our understanding of the effects of these factors during in vitro testing. Finding alternative and safe-to-swallow foods is important for improved dysphagia management. Clinical evaluation of swallowing using FEES or VFFS is cumbersome and invasive. The quantitative evaluations of the in vitro swallowing tests provides a non-invasive way to screen foodstuffs for suitability whilst reducing the number of clinical investigations and improving our understanding of how we can prepare safer foods for patients with dysphagia [41].

## Conclusions

The results of the study have answered the four stated objectives. The in vitro swallowing characteristics of thickeners, jellies and yoghurt were assessed using quantitative analysis of the in vitro-OTT and BL at the juncture of the pharynx and larynx using a throat model simulator. We found that increasing thickener content increased the in vitro-OTT and decreased BL. Xanthan gum-based thickeners showed shorter BL than starch-based thickeners when used at a low consistency. Jellies and yoghurt had similar in vitro swallowing behaviours to thickeners at high consistency. The in vitro swallowing data showed some good correlation with the reported in vivo data. However, the study also highlighted a number of discrepancies between the in vitro test conditions and the physiological swallowing process, which means caution is needed when extrapolating the in vitro data to in vivo swallowing safety. Our findings provide further evidence for using the in vitro simulator to help in the design of new thickening agents and the selection of alternative and palatable safe-to-swallow foods for dysphagia patients.

## Electronic supplementary material

Below is the link to the electronic supplementary material.

Supplementary file1 (DOCX 176 kb)
