# Supplementary material for: The Swallowing Characteristics of Thickeners, Jellies and Yoghurt Observed Using an In Vitro Model
Source: Dysphagia. 2019 Nov 9;35(4):685–95. doi: 10.1007/s00455-019-10074-1 (PMC7351813; doi:10.1007/s00455-019-10074-1)
Supplement: Supplementary file 1 — Supplementary file1 (DOCX 176 kb) [file 455_2019_10074_MOESM1_ESM.docx]

**Supplementary Materials**


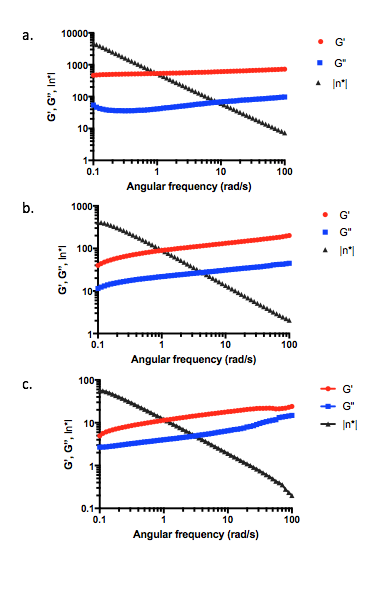


Figure 1: Oscillattory frequency sweeps for a. Thick & Easy; b. Nutilis; c. Resource Clear at stage 3 thickening.


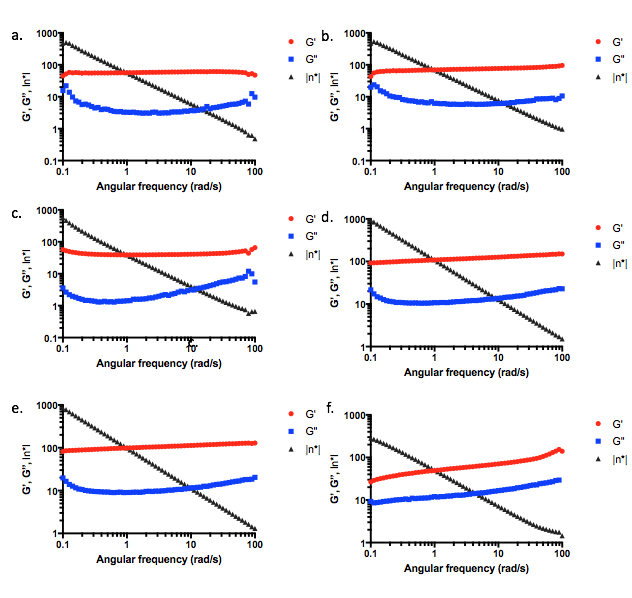


Figure 2: Oscillatory frequency sweeps for (a) Hartley’s jelly, (b) Vimto jelly, (c) Peppa pig (gelatin-based) jelly, (d) Ryukakusan jelly for adults, (e) Ryukakusan jelly for paediatrics and (f) Ski yogurt.
